# Isolation and Characterization of Environmental Extended Spectrum β-Lactamase-Producing *Escherichia coli* and *Klebsiella pneumoniae* from Ouagadougou, Burkina Faso

**DOI:** 10.3390/ph17030305

**Published:** 2024-02-27

**Authors:** Alix Bénédicte Kagambèga, René Dembélé, Oumar Traoré, Abdoul Aziz Wane, Alassane Halawen Mohamed, Hiliassa Coulibaly, Cheikh Fall, Léa Bientz, Fatima M’Zali, Laure Mayonnove, Nicolas Barro, Véronique Dubois, Yakhya Dieye

**Affiliations:** 1 Laboratoire de Biologie Moléculaire d’Epidémiologie et de Surveillance des Bactéries et Virus Transmissibles par les Aliments (LaBESTA), Université Joseph KI-ZERBO, Ouagadougou 03 BP 7021, Burkina Faso; oumar.traore@univ-dedougou.bf (O.T.); mohamed.alassane@outlook.fr (A.H.M.); coulhiliassa@gmail.com (H.C.); nicolas.barro@ujkz.bf (N.B.); 2 Unité de Formation et de Recherche en Sciences et Technologies Appliquées, Université de Dédougou, Dédougou 03 BP 176, Burkina Faso; 3 Pôle de Microbiologie, Institut Pasteur de Dakar, 36 Avenue Pasteur, Dakar BP220, Senegal; abdoul.wane@pasteur.sn (A.A.W.); cheikh.fall@pasteur.sn (C.F.); yakhya.dieye@pasteur.sn (Y.D.); 4 Laboratoire de Microbiologie de l’Hôpital Général de Référence (HGR), Niamey 8001, Niger; 5 UMR 5234 Université de Bordeaux, Faculty of Pharmacy—CNRS, Microbiologie Fondamentale et Pathogénicité, 33000 Bordeaux, France; lea.bientz@u-bordeaux.fr (L.B.); fatima.mzali@u-bordeaux.fr (F.M.); laure.coulange@u-bordeaux.fr (L.M.); veronique.dubois@u-bordeaux.fr (V.D.); 6 Groupe de Recherche Biotechnologies Appliquée et Bioprocédés Environnementaux (GRBA-BE), École Supérieure Polytechnique, Université Cheikh Anta Diop, Dakar BP 5085, Senegal

**Keywords:** AMR, ESBL-*E. coli*, ESBL-*K. pneumoniae*, municipal wastewater, stream water, organic manure, Ouagadougou

## Abstract

Antimicrobial resistance (AMR) is a global public health threat. Quality data are needed to address the rise of multidrug-resistant clones, particularly in sub-Saharan Africa. In this study, we analysed the prevalence, antimicrobial resistance profile, and presence of genes encoding extended-spectrum β-lactamase-producing *Escherichia coli* (ESBL-*Ec*) and *Klebsiella pneumoniae* (ESBL-*Kp*) in environmental samples from Ouagadougou, Burkina Faso. Of 264 samples collected, 95 (36%) and 74 (28%) contained ESBL-*Kp* and ESBL-*Ec*, respectively. ESBL-*Kp* was more prevalent in runoff water and in treated and untreated wastewater, while ESBL-*Ec* was more prevalent in manure. Interestingly, wastewater treatment did not significantly reduce the recovery of ESBL bacteria. As expected, resistance to third- and fourth-generation cephalosporins was predominant, and rare for second generation cefoxitin. Interestingly, all the isolates from treated wastewater were susceptible to ampicillin and piperacillin, while all the other clones were resistant to these antibiotics. Regarding the ESBL-encoding genes, the *bla*_CTX-M_ family was the most abundant, with the *bla*_CTX-M1_ subfamily being the most prevalent. Carriage of combinations of ESBL genes was common, with the majority of the isolates harbouring 2–4 different genes. This study highlights the need for active surveillance to manage the risk of exposure to ESBL bacteria in Burkina Faso.

## 1. Introduction

The increasing prevalence of antimicrobial resistance (AMR) is a global public health threat [1]. It is estimated that by 2030, infections caused by AMR will become a major threat to the global economy if no action is taken [2]. Low- and middle-income countries (LMICs), especially in sub-Saharan Africa, are particularly affected. A systematic analysis of the burden of AMR for 2019 showed that most deaths and disabilities directly due to or associated with AMR will occur in sub-Saharan Africa, with West Africa being the most affected region [3]. Sub-Saharan Africa suffers from several weaknesses in the fight against AMR, the most important being a lack of properly equipped bacteriology laboratories and a shortage of well-trained bacteriologists to perform accurate antimicrobial susceptibility testing and characterise bacterial pathogens [4]. This can contribute to the spread of antimicrobials and their residues in the environment, thus contributing to the selection of resistant bacteria, including human and animal pathogens. Livestock production is a major user of antimicrobials in sub-Saharan Africa due to the many infectious diseases (diarrhoea, cough, skin diseases) to which livestock are exposed [5]. Indeed, data on antimicrobial consumption in animals in Burkina Faso were highlighted in studies by Bako (2019) [6] and Ouédraogo et al. (2017) [7]. Overall, almost half of these antimicrobials belonged to the tetracycline family—which remains the most widely used in veterinary medicine worldwide (35.6% of the total)—and the penicillin family (13.3% of the total) [8]. These two antimicrobial families are included in a list of important antibiotics in veterinary medicine, which is updated regularly [9]. Of the antimicrobials on this list, fluoroquinolones and third- and fourth-generation cephalosporins account for only 3.4% and 0.6%, respectively, of the total quantities reported [8]. Wastewater and sewage systems are other major sources of environmental contamination in sub-Saharan Africa. However, wastewater treatment plants (WWTPs) are an essential link in the water quality chain. They aim to reduce the harmfulness of wastewater by biological and/or physico-chemical means before it is discharged into the natural environment. WWTPs treat the wastewater collected and produce purified water that is discharged into the natural environment, as well as purification residues known as sludge [10]. They are places where wastewater containing microorganisms, chemical residues, and nutrients from different sources such as hospitals, municipalities, industry, and agriculture, interact [11]. As a result, wastewater treatment plants are hotspots for the selection of resistant bacteria and the horizontal exchange of genetic determinants of resistance [11,12]. Conventional wastewater treatment plants only have the capacity to reduce bacteria by a factor of 10 to 100, which slows the spread of bacteria via treated water [13]. In addition, the use of treated wastewater as a fertiliser for vegetable crops is common in sub-Saharan Africa. The extent to which resistant bacteria are selected in wastewater treatment plants and the mechanisms by which they are selected remain to be elucidated, and detailed environmental studies are needed to assess the associated risks [14].

β-lactams are the most widely used antibiotics in human and veterinary medicine [15,16]. They comprise four classes, including penicillins, cephalosporins, monobactams, and carbapenems that are efficient treatments against many bacterial pathogens, including both Gram-positive and Gram-negative species. Resistance to β-lactams is primarily mediated by β-lactamases (BLs), which are enzymes that inactivate β-lactams [15]. These enzymes constitute a large and diverse family of molecules for which two classifications are proposed. The Ambler classification, based on the amino acid sequences, divides BLs in four classes named A–D [17]. The other classification groups BLs in three classes according to their substrate and inhibitor profiles [18]. Extended-spectrum β-lactamases (ESBLs) were originally defined as BLs effective against third- and fourth-generation (extended-spectrum) cephalosporins and monobactams, and inhibited by β-lactamase inhibitors like clavulanic acid, sulbactam, and tazobactam [19]. The first known ESBL are class A BLs that derive from TEM and SHV families that emerged in the 1970s from point mutations in the genes coding for the original enzymes that have a narrow spectrum [20]. In the 1980s, there emerged CTX-M type ESBL, a new subgroup of class A BLs that rapidly disseminated worldwide and constitute today the most prevalent ESBL [21]. Besides class A enzymes, which are the most prevalent, other ESBLs belong to classes C and D of the Ambler classification [22]. Interestingly, derivatives of TEM- and SHV-type ESBL that are resistant to the ancient inhibitors’ clavulanic acid, sulbactam and tazobactam emerged, challenging the initial definition of this BL family [22]. Today, ESBL, especially class A enzymes, have widely disseminated across the globe, likely because they are mostly encoded by genes located on plasmids or other mobile genetic elements [22]. There is increasing talk of establishing a link between what is observed in human medicine and what is observed in animals. But what about the likely link between effluents and the spread of ESBL bacteria in the environment? And what about the probable link between organic manure and the spread of *Enterobacterales* ESBL in the environment? Studies carried out in Burkina Faso by Bako et al. (2017) [23] and Garba et al. (2023) [24] have highlighted the use of antimicrobials in animal production and their release into the environment. Thus, we were interested in the environmental milieu of Burkina Faso, in particular its possible contamination by resistant bacteria such as *E. coli* and *K. pneumoniae*, producing extended-spectrum β-lactamases (ESBLs).

Extended-spectrum β-lactamase-producing *Enterobacterales* including *E. coli* and *K. pneumoniae* are an important public health threat in the global context of the increase of AMR [21]. *Enterobacterales* resistant to third generation cephalosporins are classified as priority 1 pathogens for which urgent actions are needed [25]. *E. coli* and *K. pneumoniae* are commensals of humans’ and animals’ gastrointestinal tract. However, these species also contain clones that are pathogenic, causing different diseases. The gastrointestinal tract is a rich ecological niche where bacteria exchange genetic material, including AMR determinants. When *Enterobacterales* are released in the environment, they contribute disseminating AMR, especially in hotspots like WWTPs. ESBL genes that are often located on plasmids are particularly concerned by this phenomenon that favours their dissemination [26]. Several studies reported ESBL *Enterobacterales* in Burkina Faso from clinical samples [27,28,29,30] and in wastewater and salads [31]. In this study, we analysed ESBL-producing *E. coli* (ESBL-*Ec*) and *K. pneumoniae* (ESBL-*Kp*) from environmental samples from Ouagadougou, capital city of Burkina Faso. We report on the prevalence, antibiotic resistance profile, and ESBL-encoding genes of isolates from runoff water, untreated and treated wastewater, and manure from two cattle markets.

## 2. Results

### 2.1. Prevalence of Extended-Spectrum β-Lactamase-Producing Escherichia coli and Klebsiella pneumoniae in Environmental Samples

During the sampling period (July 2021–April 2022), we collected 264 samples (Table 1). Additionally, in order to have an insight on the efficacy of wastewater treatment at the municipal plant, we collected samples before (*n* = 41) and after (*n* = 41) treatment (Table 1). Of the 264 samples collected, 74 (28.0%) and 95 (36.0%) contained ESBL-*E. coli* (ESBL-*Ec*) and ESBL-*K. pneumoniae* (ESBL-*Kp*), respectively, resulting in a prevalence of 64% containing one of these two bacteria (Table 1). Notably, no sample contained both species together. Not surprisingly, wastewaters were the most contaminated, while runoff waters were the least contaminated samples.

ESBL-*Kp* was significantly (*p*-value < 0.05) more prevalent than ESBL-*Ec* in runoff water and in treated and untreated wastewater. In contrast, ESBL-*Ec* was more frequently recovered than ESBL-*Kp* in manures from both livestock markets (Table 1). Surprisingly, the prevalence of ESBL-*Ec* and ESBL-*Kp*, as well as the percentage of contaminated samples, did not significantly differ between treated and untreated wastewater (Table 1), suggesting that the treatment applied to wastewater did not contribute to eliminate ESBL-producing *Enterobacterales*. Regarding single species prevalence, ESBL-*Kp* was highly prevalent in raw (68.3%) and treated (58.5%) wastewater, and was less present in runoff water (34.5%) and manure. In contrast, ESBL-*Ec* presence was highest in manure and lowest in runoff water (Table 1). Finally, it has to be noted that the prevalence of both species and of overall contaminated samples did not significantly (*p*-value > 0.05) differ between the two markets included in this study.

### 2.2. Resistance Profile of Environmental Extended-Spectrum β-Lactamase-Producing Escherichia coli and Klebsiella pneumoniae Isolates

A total of 169 isolates, including 74/169 (28%) and 95/169 (36%) *E. coli* and *K. pneumoniae*, respectively, were recovered from the environmental samples we analysed (Table 2). All isolates were tested for susceptibility to 16 antibiotics belonging to seven classes (Supplementary Tables S1 and S2). As expected, resistance to β-lactam antibiotics was common, with the highest level of resistance being to ceftriazone, a third-generation cephalosporin, to which 98.6% and 95.8% of *E. coli* and *K. pneumoniae* isolates, respectively, were resistant (Table 2). Resistance to the third-generation cephalosporin ceftazidime and the fourth-generation cephalosporin cefepime was also high in both species (Table 2). In contrast, only 1/74 (1.4%) and 7/95 (7.4%) of *E. coli* and *K. pneumoniae* isolates, respectively, were resistant to cefoxitin, a second-generation cephalosporin (Table 2). As for resistance to other β-lactams, it was common for ampicillin and piperacillin, which belong to the penicillin subfamily, and aztreonam (a monobactam), while it was rare in the carbapenem subfamily, with all isolates tested susceptible to imipenem and 5.4% and 7.4% of *E. coli* and *K. pneumoniae* clones, respectively, resistant to ertapenem (Table 2). Interestingly, 17.9% and 8.1% of *K. pneumoniae* and *E. coli* isolates, respectively, were resistant to the amoxicillin/clavulanic acid combination (Table 2), confirming the increasing frequency of inhibitor-resistant ESBL enzymes in *Enterobacterales*, which has led to the development of new ESBL inhibitors like tazobactam, to which only one clone of each species was resistant (Table 2). Apart from β-lactams, resistance to the other families of antibiotics tested (quinolones, fluroquinolones, sulphonamides, and aminoglycosides) was comparable in both species with the exception of nalidixic acid (quinolones family) to which 43.2% and 9.5% of *E. coli* and *K. pneumoniae* isolates, respectively, were resistant (Table 2). Resistance was high for the sulfamethoxazole-trimethoprim combination (anti-folates family), moderate for ciprofloxacin, and rare for the aminoglycosides gentamicin and amikacin (*p* value < 0.5) (Table 2). When analysing resistance according to the origin of the isolates, we strikingly found that all the clones from treated wastewater, regardless of the species, were susceptible to the penicillins ampicillin and piperacillin, while all the other bacteria were resistant to these antibiotics (Supplementary Tables S1 and S2). Additionally, the 6/8 isolates resistant to cefoxitin were *K. pneumoniae* clones (Supplementary Tables S1 and S2).

### 2.3. Detection of Extended-Spectrum β-Lactamase-Encoding Genes

**Figure 1:**
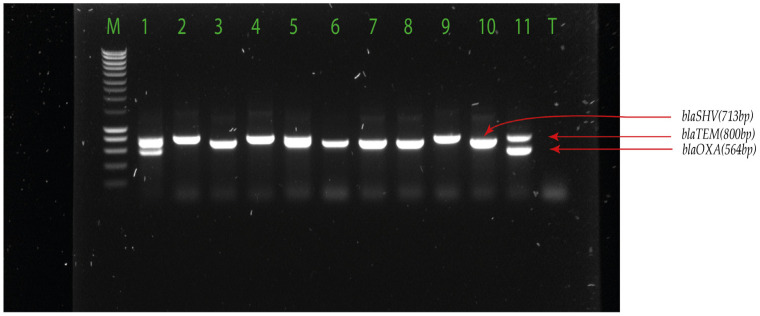
*bla*_TEM_, *bla*_SHV_, *bla*_OXA_ detected in *E. coli* and *K. pneumoniae*. Legend: M = hyperlader 100 pb; 1 = *bla*_*SHV*-OXA_ positive samples; 5 = *bla*_TEM-SHV_ positive samples; 3, 6, 7 and 8 = *bla*_SHV_ positive samples (713 pb); 2 and 4 = *bla*_TEM_ positive samples (800 pb); 9 = *bla*_TEM_ positive control (800 pb); 10 = *bla*_SHV_ positive control (713 pb); 11 = *bla*_OXA_ positive control (564 pb); T: negative control.

**Figure 2:**
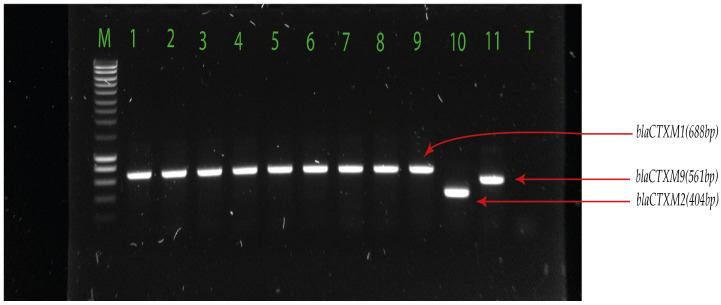
*bla*_CTX-M-1_, *bla*_CTX-M-2_, and *bla*_CTX-M-9_ detected in *E. coli* and *K. pneumoniae*. Legend: M = hyperlader 100 pb; 1 to 8 = *bla*_CTX-M-1_ positive samples (688 pb); 9 = *bla*_CTX-M-1_ positive control (688 pb); 10 = *bla*_CTX-M-2_ positive control (404 pb); 11 = *bla*_CTX-M-9_ positive control (561 pb); T = negative control.

We used PCR to detect ESBL-encoding genes, including *bla*_CTX-M_ (Figure 1)*, bla*_TEM_, *bla*_SHV_, and *bla*_OXA_ (Figure 2) families in the isolates (Table 3 and Supplementary Tables S1 and S2).

As expected, all the clones with the exception of one *E. coli* had at least one ESBL gene (Table 3)*. bla*_CTX-M_ genes were the most prevalent, with *bla*_CTX-M1_ detected in 85.1% and 93.7% of the *E. coli* and *K. pneumoniae* isolates, respectively (Table 3). In contrast, *bla*_CTX-M9_ genes was detected in 10.8% and 3.2% *E. coli* and *K. pneumoniae* isolates respectively, while *bla*_CTX-M2_ was not found in any isolate (Table 3). These results are consistent with the worldwide spread of *bla*_CTX-M_ ESBL genes reported in studies by other authors [32]. Besides *bla*_CTX-M_ family, the most frequent ESBL gene was *bla*_TEM_ whose presence in the two species was compared using statistical tests (Pearson χ^2^, 3.494, *p* = 0.062) (Table 3). Similarly, distribution of *bla*_OXA_ genes was comparable in the two bacteria. In contrast, *bla*_SHV_ were significantly more frequent in *K. pneumoniae* than in *E. coli* (Pearson χ^2^, 41.480, *p* < 0.001) (Table 3). Notably, no isolate contained a *bla*_OXA_ gene in the absence of another ESBL gene (Table 4). Carriage of combinations of ESBL genes was frequent, with 58.1% (43/74) and 57.9% (55/95) of *E. coli* and *K. pneumoniae* isolates, respectively, harbouring more than one of these genes (Table 4). The most frequent combination was *bla*_CTX-M_*-bla*_TEM_ in *E. coli* and *bla*_CTX-M_*-bla*_SHV_ in *K. pneumoniae* (Table 4). A significant proportion of isolates in both species carried three ESBL genes, and four *K. pneumoniae* clones contained the four families of ESBL genes analysed (Table 4).

## 3. Discussion

In this study, we isolated and characterized ESBL-*Ec* and ESBL-*Kp* isolates from environment samples in Ouagadougou, Burkina Faso. Not surprisingly, both species were present in all types of samples, with ESBL-*Kp* more prevalent in runoff water and wastewater, while ESBL-*Ec* was more frequent in manure. The isolates displayed resistance to several β-lactam antibiotics, as well as to other antimicrobial families. Additionally, we detected the presence of ESBL genes supporting the resistance phenotype we observed. This study gives important insights on the prevalence and dissemination of ESBL-*Ec* and ESBL-*Kp* in the environment in Ouagadougou, Burkina Faso. *E. coli* and *K. pneumoniae* are important human and animal pathogens. Both species comprise gut commensals and pathogenic clones. Importantly, they are ubiquitous, making them efficient vehicles for AMR dissemination. The presence of ESBL-*Ec* and ESBL-*Kp* in rainwater runoff could be explained by the marshy nature of the city of Ouagadougou, which is crossed by the Massili slope and many watercourses that potentially contain ESBL-*Enterobacterales* originating from the excrement of grazing cattle or of wild animals, discharge of partially treated wastewater, or sewer overflows during heavy rains [33,34]. These observations point to the need to conduct additional studies in order to evaluate the risks of these potential contamination sources and mitigate their occurrence. In this regard, the high prevalence of ESBL-*Ec* found in manure is compelling. *E. coli* is commonly a predominant species in stools from humans and animals. The high prevalence of ESBL-*Ec* might indicate a high usage of antibiotics for cattle. Antibiotics are widely used in animal husbandry, and various types of antibiotic resistance genes (ARGs) are frequently detected in livestock waste around the world. For instance, in 2017, the global consumption of antibiotics in chicken, cattle, and pigs was estimated to be 93,309 t of the active ingredients [35]. This practice is well known to be a cause of selection and dissemination of resistant bacteria and of AMR genes. These antimicrobials are used as a growth promoter, and in the absence of vaccines for most of the infectious diseases that affect cattle in sub-Saharan Africa, farmers heavily use antimicrobials as prophylactics. In high-income countries, this concern is properly addressed by legislation and regulations applied to food manufacture and commercialization [36]. In contrast, such regulations do not exist in many low-income countries, or when they exist, they are often not enforced. Indeed, it is known that animals and farm environments are important reservoirs of ESBL-producing bacteria [37,38]. In addition, animal waste carrying ESBLs lead to biological contamination and accumulation in water and soil [39,40,41].

Interestingly, we found that the prevalence of both ESBL-*Ec* and ESBL-*Kp* did not significantly differ between treated and untreated samples from the municipal wastewater treatment plant. This result was surprising, since the treatment procedure at urban wastewater plants typically includes the use of disinfectants that dramatically reduce the bacterial load and prevent the release of pathogenic bacteria into the environment [42]. Indeed, treated municipal wastewater is commonly discharged in natural water bodies or used as fertilizer for gardening and agriculture [43]. However, since we did not measure the bacterial load of the wastewater samples we analysed, but rather applied direct selection of ESBL bacteria, we cannot conclude on the efficacy of the treatment procedure at the Ouagadougou municipal treatment plant. Additionally, wastewaters are known to constitute AMR hotspots characterized by high densities of intact antimicrobial molecules that are not fully metabolized, antimicrobial residues, resistant bacteria, and AMR genes (ARGs) [44]. WWTPs are places where wastewater containing micro-organisms, chemical residues, and nutrients from different sources such as hospitals, communities, industry, and agriculture interact [11]. Consequently, wastewater treatment plants represent hotspots for the selection of resistant bacteria and the horizontal exchange of genetic determinants of resistance [11,12]. Such an environment therefore favours the spread of AMR and the selection of resistant bacteria by various mechanisms, in particular the expression of ARGs induced by antimicrobials and the horizontal transfer of ARGs by conjugation, transduction, and natural competition [45]. Several studies reported wastewater treatment that only slightly reduced the load of resistant bacteria [46]. The fact that 7/8 of the cefoxitin-resistant isolates we analysed came from treated wastewater (Table 2, Supplementary Tables S1 and S2) might reflect a selection even though the number of clones is too low to be significant.

Another important observation we made was the apparent selection of clones susceptible to penicillins (ampicillin and piperacillin) in treated wastewater where all the strains displaying this phenotype were found (Supplementary Tables S1 and S2). We do not have an explanation for this observation. Noticeably, all the isolates showed the same phenotype for ampicillin and piperacillin (resistant or susceptible to both), suggesting that the same gene confers resistance to both molecules. It might be that the expression of this gene results in the activation of mechanisms pertaining to components of the wastewater treatment that lead to bacterial killing. However, we do not have any evidence that supports this hypothesis, which needs to be verified.

## 4. Materials and Methods

### 4.1. Study Design, Sites, and Sampling

**Figure 3:**
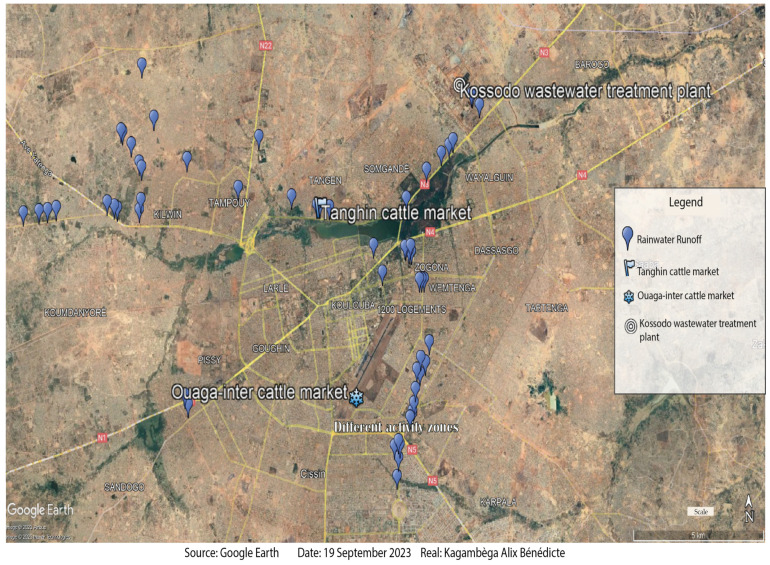
Sampling sites.

To assess the level of dissemination of ESBL-*Ec* and ESBL-*Kp* in the environment in Ouagadougou (12°21′56.4″ N, 1° 32′2″ W), Burkina Faso, we conducted a prospective sampling of runoff water (58 samples), wastewater from the municipal treatment plant (82 samples), and manure (124 samples) from two cattle markets, which are livestock collection, assembly, and consumption markets (Figure 3). Sampling was carried out over a 10-month period between July 2021 and April 2022 in order to cover as many seasons of the year as possible. WWTP effluent and run-off water, which are prone to pollution, were sampled in 250 mL sterile glass vials. Instantaneous samples were taken upstream (untreated wastewater) and downstream (treated wastewater) of the WWTP, which is the only plant treating urban wastewater. Once in the laboratory, these samples were processed in the same way. Sampling of organic manure consisted of collecting approximately 200 g of the manure using a sterile spatula. The weight of the sample was measured using a portable digital scale. The samples were then placed in sterile plastic bags and sent to the laboratory for processing.

### 4.2. Microbial Culturing and Identification

For bacterial isolation, 10 mL of wastewater, 10 mL of effluent, or 10 g of organic manure were each serially diluted in 90 mL of sterile buffered peptone water. For the isolation of ESBL bacteria, sample dilutions were used to inoculate coliform Chromocult agar (CCA) (Laboratorios Conda S.A; Torrejón d’Ardoz, Spain) or plate count agar (PCA) + triphenyl tetrazolium chloride (TTC) agar supplemented with 2 mg/L ceftriaxone. Agar was incubated at 37 °C for 24 h. Presumptive colonies on Coliform Chromocult Agar, *E. coli* positive colonies take on a dark blue to purple colour. These colonies are easily distinguished from other coliform colonies, which are pink to red in colour. On plate count agar, presumptive *E. coli* and *K. pneumoniae* are characterised by a red to brown colour.

Presumptive colonies were then streaked onto eosin methylene blue (EMB) agar (Laboratorios Conda S.A) and incubated at 37 °C for 24 h for isolate purification and preservation.

Muller Hinton (MH) agar (Laboratorios Conda S.A.) was used to subculture the isolates at 37 °C ± 0.5 °C for 24 h, and identification was performed by MALDI-TOF (Matrix-Assisted Laser Desorption-Ionization-Time Of Flight) mass spectrometry using the Microflex MALDI-TOF MS^®^ instrument (Bruker Daltonics, Bremen, Germany). To avoid confusion with Shigella, *E. coli* isolates identified by MALDI-TOF were confirmed on a BromoCresol Purple (BCP) agar plate and subsequently on an API 20E gallery (bioMerieux, Marcy-l’Etoile, France).

### 4.3. Antibiotic Susceptibility Testing

Antimicrobial susceptibility testing against 16 antibiotics was performed using the agar disc diffusion method on MH agar plates, according to the guidelines of the European Committee on Antimicrobial Susceptibility Testing (EUCAST) 2021 [47]. They were then incubated at 37 °C for 24 h. The following antibiotics were used (MAST, Amiens, France): Ampicillin 10 μg/mL, piperacillin 30 μg/mL, piperacillin-tazobactam 36 μg/mL, amoxicillin-clavulanic acid 30 μg/mL, aztreonam 30 μg/mL, imipenem 10 μg/mL, ertapenem 10 μg/mL, ceftriaxone 30 μg/mL, cefoxitin 30 μg/mL, cefepime 30 μg/mL, ceftazidime 10 μg/mL, gentamicin 10 μg/mL, amikacin 30 μg/mL, nalidixic acid 30 μg/mL, ciprofloxacin 5 μg/mL, and sulphamethoxazole-trimethoprim 25 μg/mL. *E. coli* ATCC 25922 and *K. pneumoniae* ATCC 700603, which produce an ESBL (SHV-18), were used for quality control.

The ESBL phenotype was confirmed for isolates grown on CCA-ceftriaxone or PCA+TTC-ceftriaxone by testing their resistance to third and fourth generation cephalosporins, including cefotaxime, ceftazidime, ceftriaxone, and cefepime. In addition, the double-disc synergy test was performed using an amoxicillin/clavulanic acid combination. A positive synergy test was recorded as confirmation of the ESBL-producing phenotype. An isolate grown on a CCA-ceftriaxone or PCA+TTC-ceftriaxone plate that was resistant to third- and fourth-generation cephalosporins and to the amoxicillin/clavulanic acid combination was considered to be an inhibitor-resistant ESBL producer.

### 4.4. Detection of ESBL Genes

DNA was extracted from *E. coli* or *K. pneumoniae* isolates using organic extraction (phenol-chloroform method) as previously described [48]. The phenol-chloroform method for DNA extraction was used because of the rapid denaturation of nucleases and stabilisation of nucleic acids. The quantity, purity, and integrity of extracted DNA were verified by 1% agarose gel electrophoresis and measurement using a NanoDrop™ spectrophotometer. Multiplex PCR was performed for detection of ESBL genes (*bla*_TEM (Temoneira patient name)_, *bla*_SHV (Sulfhydryl variable)_, *bla*_OXA (oxacillinase)_, *bla*_CTX-M1_, *bla*_CTX-M2_, and *bla*_CTX-M9_*)* using primers previously described (Table 5). Two multiplex PCRs were performed: one detecting *bla*_TEM_/*bla*_SHV_/*bla*_OXA_, and another targeting *bla*_CTX-M (Cefotaximase-Munich)_ subgroups 1, 2, and 9. Five (5) μL of sample DNA was subjected to each multiplex PCR in a 25 μL reaction mixture containing a PCR buffer (5X), primers (10 µM), and PCR water, using the GoTaq^®^ Master mixes (Promega, Charbonnières-les-Bains, France). The amplification of the ESBL genes was carried out as follows: initial denaturation at 95 °C for 3 min; 34 cycles of 95 °C for 40 s, 60 °C for 40 s, 72 °C for 1 min, and a final elongation step at 72 °C for 5 min. Amplicons were visualized on a 1% ethidium bromide agarose gel. For quality control, we used clinical strains obtained from the Pelegrin hospital of Bordeaux for which the genes have been sequenced [48].

### 4.5. Statistical Analyses

Statistical analyses were performed using IBM SPSS v29 software. Pearson Chi-Square was used to compare the prevalence of ESBL-*Ec* and ESBL-*Kp* in the different types of samples, as well as the frequency of ESBL genes between *E. coli* and *K. pneumoniae* with a *p* value < 0.05 being considered as statistically significant.

## 5. Conclusions

In this study, we detected a high prevalence of ESBL-*Ec* and ESBL-*Kp* in environmental samples from Ouagadougou, Burkina Faso. Our findings show an important risk of acquisition, from the environment, of the bacteria that comprise clones that cause clinical infections in humans and animals. Given the importance of β-lactams for the treatment of bacterial infections, our results stress the need for active surveillance of AMR to monitor the dissemination of the risk of acquisition of multi-resistant bacteria from the environment. Additionally, our results revealed a possible selection of bacteria at the municipal wastewater treatment plant that needs to be investigated in order to understand its mechanisms and prevent dissemination of AMR from this important site of horizontal transfer of genetic determinants of resistance.

## Figures and Tables

**Table 1:** Prevalence of ESBL-*E. coli* (ESBL-*Ec*) and ESBL-*K. pneumoniae* (ESBL-*Kp*) in environmental samples *.

Sample Type		*E. coli*-Positive	*K. pneumoniae*-Positive	Negative Samples	Total
Runoff water		3 (5.2%)	20 (34.5%)	35 (60.3%)	58
Wastewater	Untreated	6 (14.6%)	28 (68.3%)	7 (17.1%)	41
	Treated	11 (26.8%)	24 (58.5%)	6 (14.6%)	41
Manure	Ouaga-Inter livestock market	30 (48.4%)	11 (17.7%)	21 (33.9%)	62
	Tanghin livestock market	24 (38.7%)	12 (19.4%)	26 (41.9%)	62
Total		74 (28.0%)	95 (36.0%)	95 (36.0%)	264

* The percentage values shown correspond to that of the corresponding sample types. ESBL: extended spectrum β-lactamase-producing; *Ec*: *Escherichia coli*; *Kp*: *Klebsiella pneumoniae*.

**Table 2:** Antibiotic resistance of environmental extended-spectrum β-lactamase-producing *Escherichia coli* and *Klebsiella pneumoniae* isolates *.

Antibiotics	*E. coli*	*K. pneumoniae*
Ampicillin	63/74 (85.1%)	71/95 (74.7%)
Piperacillin	63/74 (85.1%)	71/95 (74.7%)
Cefoxitin	1/74 (1.4%)	7/95 (7.4%)
Ceftazidime	49/74 (66.2%)	67/95 (70.5%)
Ceftriaxone	73/74 (98.6%)	91/95 (95.8%)
Cefepime	38/74 (51.4%)	64/95 (67.4%)
Aztreonam	50/74 (67.6%)	62/95 (65.3%)
Ertapenem	4/74 (5.4%)	7/95 (7.4%)
Imipenem	0	0
Amoxicillin-Clavulanic acid	6/74 (8.1%)	17/95 (17.9%)
Piperacillin-Tazobactam	1/74 (1.4%)	1/95 (1.5%)
Gentamicin	6/74 (8.1%)	7/95 (7.4%)
Amikacin	1/74 (1.4%)	0
Nalidixic acid	32/74 (43.2%)	9/95 (9.5%)
Ciprofloxacin	24/74 (32.4%)	33/95 (34.7%)
Sulfamethoxazole-Trimethoprim	44/74 (59.5%)	66/95 (69.5%)

* The numbers correspond to isolates with resistant ^®^ phenotype.

**Table 3:** Presence of extended spectrum β-lactamase encoding genes in environmental ESBL-producing *Escherichia coli* and *Klebsiella pneumoniae*.

Genes	*E. coli*	*K. pneumoniae*
*bla*_TEM_	33/74 (44.6%)	28/95 (29.5%)
*bla*_SHV_	7/74 (9.5%)	56/95 (58.9%)
*bla*_OXA_	10/74 (13.5%)	10/95 (10.5%)
*bla*_CTX-M1_	63/74 (85.1%)	89/95 (93.7%)
*bla*_CTX-M9_	8/74 (10.8%)	3/95 (3.2%)
*bla*_CTX-M2_	0	0

The numbers correspond to isolates harbouring the respective *ESBL* genes. *ESBL*, extended spectrum β-lactamase-producing.

**Table 4:** Single or combination of extended spectrum β-lactamase encoding genes carried by environmental ESBL-producing *Escherichia coli* and *Klebsiella pneumoniae*.

Genes	*E. coli*	*K. pneumoniae*
*bla*_CTX-M_-*bla*_TEM_-*bla*_SHV_-*bla*_OXA_	0	4/95 (4.2%)
*bla*_CTX-M_-*bla*_TEM_-*bla*_SHV_	2/75 (2.7%)	10/95 (10.5%)
*bla*_CTX-M_-*bla*_TEM_-*bla*_OXA_	2/75 (2.7%)	2/95 (2.1%)
*bla*_CTX-M_-*bla*_SHV_-*bla*_OXA_	1/75 (1.3%)	3/95 (3.2%)
*bla*_CTX-M_-*bla*_SHV_	3/75 (4%)	36/95 (37.9%)
*bla*_CTX-M_-*bla*_TEM_	27/75 (36%)	11/95 (11.6%)
*bla*_CTX-M_-*bla*_OXA_	7/75 (9.3%)	1/95 (1.1%)
*bla*_TEM_-*bla*_SHV_	1/75 (1.33%)	1/95 (1.1%)
*bla*_CTX-M_	29/75 (38.7%)	25/95 (26.3%)
*bla*_SHV_	0	2/95 (2.1%)
*bla*_TEM_	1/75 (1.3%)	0
No ESBL gene	1/75 (1.3%)	0

*bla*_CTX-M_ refers to isolates carrying *bla*_CTX-M1_ or *bla*_CTX-M9_. %: Prevalence rate of isolates containing one or more genes.

**Table 5:** List of primers used for the assays.

ESBL Target Genes	Primer Sequences (5′-3′)	Amplicon Size (bp)	Reference
TEM	F: CATTTCCGTGTCGCCCTTATTC	800	[49]
	R: CGTTCATCCATAGTTGCCTGAC		[49]
SHV	F: AGCCGCTTGAGCAAATTAAAC	713	[49]
	R: ATCCCGCAGATAAATCACCAC		[49]
OXA	F: GGCACCAGATTCAACTTTCAAG	564	[49]
	R: GACCCCAAGTTTCCTGTAAGTG		[49]
CTX-M1	F: TTAGGAARTGTGCCGCTGYA ^b^	688	[49]
	R: CGATATCGTTGGTGGTRCCAT ^b^		[49]
CTX-M2	F: CGTTAACGGCACGATGAC	404	[49]
	R: CGATATCGTTGGTGGTRCCAT ^b^		[49]
CTX-M9	F: TCAAGCCTGCCGATCTGGT	561	[49]
	R: TGATTCTCGCCGCTGAAG		[49]

^b^ R = A or G.

## Data Availability

The data presented in this study are available upon request to the corresponding author.

## Supplementary Materials

The following supporting information can be downloaded at: https://www.mdpi.com/article/10.3390/ph17030305/s1, Table S1. Origin, resistance profile and presence of ESBL genes in environmental *E. coli* isolates; Table S2. Origin, resistance profile and presence of ESBL genes in environmental *K. pneumoniae* isolates.
